# Preregistration of animal research protocols: development and 3-year overview of preclinicaltrials.eu

**DOI:** 10.1136/bmjos-2021-100259

**Published:** 2022-03-16

**Authors:** Mira van der Naald, Steven A J Chamuleau, Julia M L Menon, Wim de Leeuw, Judith de Haan, Dirk J Duncker, Kimberley Elaine Wever

**Affiliations:** 1 Department of Cardiology, University Medical Center Utrecht, Utrecht, The Netherlands; 2 Department of Cardiology, Amsterdam UMC Locatie AMC, Amsterdam, North Holland, The Netherlands; 3 Netherlands Heart Institute, Utrecht, The Netherlands; 4 Animal Welfare Body Utrecht, Utrecht, The Netherlands; 5 Open Science Programme, Utrecht University, Utrecht, The Netherlands; 6 Department of Cardiology, Thoraxcenter, Erasmus Medical Center, Rotterdam, Zuid-Holland, The Netherlands; 7 Systematic Review Centre for Laboratory Animal Experimentation (SYRCLE), Department for Health Evidence, Radboud University Medical Center, Nijmegen, The Netherlands; 8 Department of Anesthesiology, Radboud University Medical Center, Nijmegen, The Netherlands

**Keywords:** biomedical research, research design

## Abstract

Open, prospective registration of a study protocol can improve research rigour in a number of ways. Through preregistration, key features of the study’s methodology are recorded and maintained as a permanent record, enabling comparison of the completed study with what was planned. By recording the study hypothesis and planned outcomes a priori, preregistration creates transparency and can reduce the risk of several common biases, such as hypothesising after results are known and outcome switching or selective outcome reporting. Second, preregistration raises awareness of measures to reduce bias, such as randomisation and blinding. Third, preregistration provides a comprehensive listing of planned studies, which can prevent unnecessary duplication and reduce publication bias. Although commonly acknowledged and applied in clinical research since 2000, preregistration of animal studies is not yet the norm. In 2018 we launched the first dedicated, open, online register for animal study protocols: wwwpreclinicaltrialseu. Here, we provide insight in the development of preclinicaltrials.eu (PCT) and evaluate its use during the first 3 years after its launch. Furthermore, we elaborate on ongoing developments such as the rise of comparable registries, increasing support for preregistration in the Netherlands—which led to the funding of PCT by the Dutch government—and pilots of mandatory preregistration by several funding bodies. We show the international coverage of currently registered protocols but with the overall low number of (pre)registered protocols.

## Disclaimer

This article expands on an existing short communication paper published in *PLoS Biology*.^1^

## Introduction

Although controversial, animal experiments are still considered essential in many fields of biomedical and toxicological research. Unfortunately, concerns are raised about their validity and robustness, especially when new therapies based on promising animal studies fail to show clinical efficacy, safety and return on investment.^2 3^ A thorough investigation of the causes of translational failure is currently hampered by the lack of rigorous science. Key requirements for highly robust experimental data are adequate statistical power, a study design which maximises external validity and high internal study validity. Furthermore, reporting on all performed experiments should be complete and transparent, regardless of their outcome. In addition, studies should be optimised by previous findings, and thus new experiments should be preceded by an evaluation of relevant literature. Unfortunately, preclinical animal studies currently show major deficits in all of these areas, causing key findings to be difficult to reproduce and translate.^4 5^ Perhaps the most important (and highly common) problem in individual animal studies is poor reporting of study methodology.^6–8^ This includes incomplete reporting of details of the study design and animal characteristics relevant to external validity, as well as measures to reduce bias and details of the statistical analysis.^9–11^ As such, poor reporting affects all key requirements and obscures the true state of affairs in animal studies, rendering external validity, internal validity and statistical robustness and power largely unclear. Meta-research shows that studies failing to report measures to reduce bias tend to report larger effect sizes, suggesting an overestimation of the true effect size due to low internal validity.^12 13^ The limitations found within studies are further exacerbated by reporting biases such as publication bias and selective outcome reporting. The publication rate of animal studies has been shown to be limited to 60%–67%,^14 15^ and especially studies yielding neutral results or results contradicting existing evidence remain unpublished.^16–19^ Simultaneously, the under-reporting of animals in publications suggests that data are reported selectively, which can cause outcome reporting bias.^14^ Finally, outcome switching and hypothesising after results are known (HARKing) are additional forms of bias that affect research. These arise when researchers deviate from their research questions and/or plans as originally set up or when no a priori plan is in place at all. Research into these forms of bias has been dependent on open access registration of clinical trial protocols and comparing them with their subsequent publications.^20^ Animal study protocols are not registered or inaccessible, and therefore hardly any evidence on outcome switching or HARKing in preclinical research exists. However, there is no reason to assume that animal research would be immune to these biases.

## Preclinicaltrials.eu (PCT): an online international register of preclinical trial protocols

Our vision is to optimise the efficacy of preclinical research for improving human health. We propose that registration of a protocol before starting an experiment (preregistration) can play an essential role in improving the robustness and transparency of animal studies and lead to more reliable research. Such preregistration of preclinical studies has four main benefits:^21 22^

1. Disclosing the a priori study intention, that is, hypothesis, exploratory or confirmatory character and key elements of its design, including primary and secondary outcomes and sample size calculations.
2. Promoting the use of methods to reduce risks of bias (ie, blinding and randomisation) and creating transparency about their use.
3. Providing a complete overview of all performed studies (including those that remain unpublished) and the possibility to share or link to related data.
4. Creating transparency and accountability within the research community and towards society.

Several other initiatives have been developed to improve animal study robustness, for example, guidelines for planning (Planning Research and Experimental Procedures on Animals: Recommendations for Excellence (PREPARE) guidelines) and reporting (Animal Research: Reporting of In Vivo Experiments (ARRIVE) guidelines). Compared with guidelines for reporting, the added value of preregistration is its timing. Requesting the ARRIVE-checklist (or any other reporting guideline) at the submission stage may improve reporting, but for that particular research project it is too late to optimise the study design.^7^ Akin to the PREPARE guidelines,^23^ preregistration supports scientists much earlier in the research process, that is, during planning and execution of the study, thereby improving research rigour and robustness. For instance, researchers who are unfamiliar with measures to reduce bias can be made aware of implementing these measures within their study protocol. Importantly, preregistration requires sharing of key elements of the proposed outcome measures and a prespecified statistical analysis plan, enabling insight in a priori versus post-hoc analyses. Compliance with preregistration can be monitored by multiple stakeholders (ie, funders, institutes, journal editors, reviewers), whereas reporting guidelines are mostly checked by reviewers only. Importantly, preregistration can reduce unnecessary repetition of animal studies, since new animal studies should be preceded by a (systematic) search to prevent repetition, help formulate relevant research questions and optimise the animal model. Similarly, consulting an animal study registry can be useful when searching for potential collaborators. Of note, study protocols are already widely used in the approval process of animal studies, although the protocol format and the level of detail required may differ per country or even per institute. However, in general, we expect most information required for registration in an animal study registry to also be included in the study’s application for local approval.

### The development of PCT

**Figure 1:**
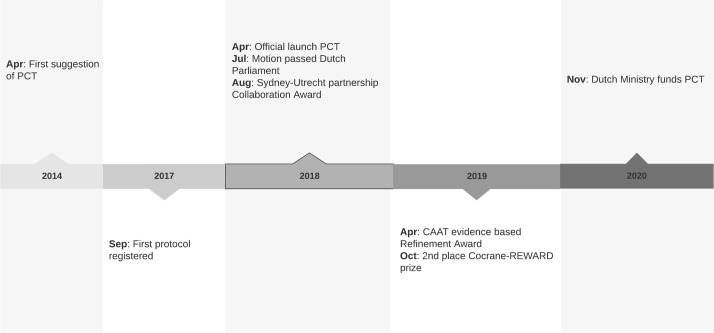
Timeline of the development of preclinicaltrials.eu (PCT).

In 2014 we first published a review suggesting an online registry for preclinical trial protocols.^24^ In the following years, we developed the first registry dedicated to animal studies to facilitate preregistration: PCT (figure 1). This initiative was developed with the help of several stakeholders to create a solid, robust base. We assembled a steering committee and attracted the Netherlands Heart Institute as an independent party responsible for hosting and reviewing submitted protocols. Subsequently, the University Medical Center Utrecht formed the legal entity. The PCT advisory board was established in 2018 to provide solicited and unsolicited advice to the steering committee regarding, for example, the future direction of the registry and the implementation of preregistration. Board members are based in various countries, various research fields and multiple disciplines, in particular (but not limited to) animal research and meta-research. Current members are Professor John Ioannidis (Stanford University, USA), Professor Jonathan Kimmelman (McGill University, Canada), Professor Paul Glasziou (Bond University, Australia), Professor Lina Badimon (IR-Hospital de la Santa Creu i Sant Pau, Autonomous University Barcelona, Spain) and Professor Thomas Eschenhagen (University Medical Center Hamburg Eppendorf, Germany). We have organised yearly meetings of the steering committee with the advisory board.

The format of the protocol registration form was discussed with fellow researchers from the Transnational Alliance for Regenerative Therapies in Cardiovascular Syndromes (TACTICS) group, the Radboud University Medical Center, University Medical Center Utrecht, the University of Sydney and several animal welfare bodies within the Netherlands. Based on this, we optimised the level of detail of the information required for registration (including which information should be mandatory vs optional) and aimed to determine the minimal amount of detail required to have an impact on research rigour, thereby minimising the additional administrative burden for researchers (a common concern regarding preregistration among researchers, see table 1). Most information required for registration would likely already be documented in a study’s experimental protocol, which is often required for approval by a local committee, as per our experience with such applications in the Netherlands. We simultaneously set out to further reduce the administrative burden for researchers by enabling an automatic transfer of the required information from local digital systems to the PCT format. After reaching out to developers of such software, this function is now in place for PRIS, a system used in several institutes in the Netherlands for animal study protocols submission to local animal welfare bodies. This allows researchers to copy most of the required information from their local application form to PCT with the click of a button. Discussions with other software developers are ongoing.

**Table 1:** Concerns often mentioned in discussions with colleagues during the development of preclinicaltrials.eu and our solutions

Concerns	Solutions
Cost	Free submission of protocol Free use of database
Administrative burden	Export data from existing study protocols
Limited flexibility of creativity	Tracked-changed adjustments are allowed
Misuse by animal activists	Login required Personal details anonymised
Data theft	Embargo
Threat to intellectual property	Embargo Time-stamped protocols

After optimising the registration form, we added functional options to the registry to overcome two other well-known concerns among researchers, namely (1) the privacy of researchers submitting protocols and (2) the risk of intellectual theft of research ideas or loss of intellectual property. Regarding privacy, personal details of the researcher submitting the protocol are anonymised, except for the institution where the experiments are performed. It is possible to contact the submitting researcher through an encrypted email message to facilitate contact and collaboration. To prevent abuse, detailed information of study protocols can only be accessed after creating an account and logging in. Without an account only limited data (titles, study centre details) of studies are visible. Regarding the fear of sharing preliminary ideas, PCT provides the option to register a protocol under embargo. The full details of the protocol remain hidden until revealed by the investigator or after a release date which is automatically set at 1 year after registration. We feel that even though an embargo delays our aim to create full transparency, the other benefits of preregistration outweigh this downside. Also, we propose that the option to register under embargo is necessary at this stage, until preregistration becomes the gold standard and the research community comes to view preregistration as a safeguard against intellectual theft of scientific ideas and intellectual property (since preregistration in fact ’claims’ an idea), rather than a risk.

### Results after 3 years of PCT

The first protocol on PCT was published in September 2017 (PCTE0000098). A position paper from TACTICS supporting PCT and discussing the importance of preregistration was published in January 2018 (figure 1).^21^ Subsequently, PCT was officially launched in April 2018, at the scientific session ‘Promoting Transparency in Preclinical Research’ held at the Netherlands Heart Institute.^25^ In November 2019, the Netherlands Heart Institute organised a round table discussion to explore possibilities to implement preregistration within the Netherlands. Over 20 participants from different universities, funders and the government were present.

The Royal Netherlands Academy of Arts and Sciences stated in 2018 that funders and journals should make preregistration mandatory for hypothesis-testing research.^26^ After the launch of PCT, the discussion on preregistration in the Netherlands intensified substantially. On 28 June 2018, members of the Dutch parliament unanimously accepted a motion stimulating preregistration for all animal research in the Netherlands.^27^ In response, the Dutch government supported the PCT initiative and in November 2020 the Dutch Ministry of Agriculture, Nature and Food quality provided funding for its maintenance and further development.^28 29^ The board of directors of the University Medical Center Utrecht agreed to stimulate preregistration of animal studies within their facilities, focusing principally on preregistration of confirmatory studies as defined by Kimmelman *et al*.^30^ Several funding agencies (including the Collaborating Health Foundations) within the Netherlands support preregistration, and the Netherlands Organisation for Health Research and Development (ZonMw) made preregistration a requirement for funding of animal studies in several pilot programmes.^31^

Since its launch, PCT has been internationally recognised for its importance in promoting rigour in animal studies. In 2018, we received the University of Sydney–Utrecht Partnership Collaboration Award, together with Dr Kieron Rooney, to empower collaboration on preregistration. In April 2019, PCT received the Science-based Refinement Award from Johns Hopkins University Center for Alternatives to Animal Testing. In August 2019, we were awarded second place in the Cochrane-REWARD prize.

**Figure 2:**
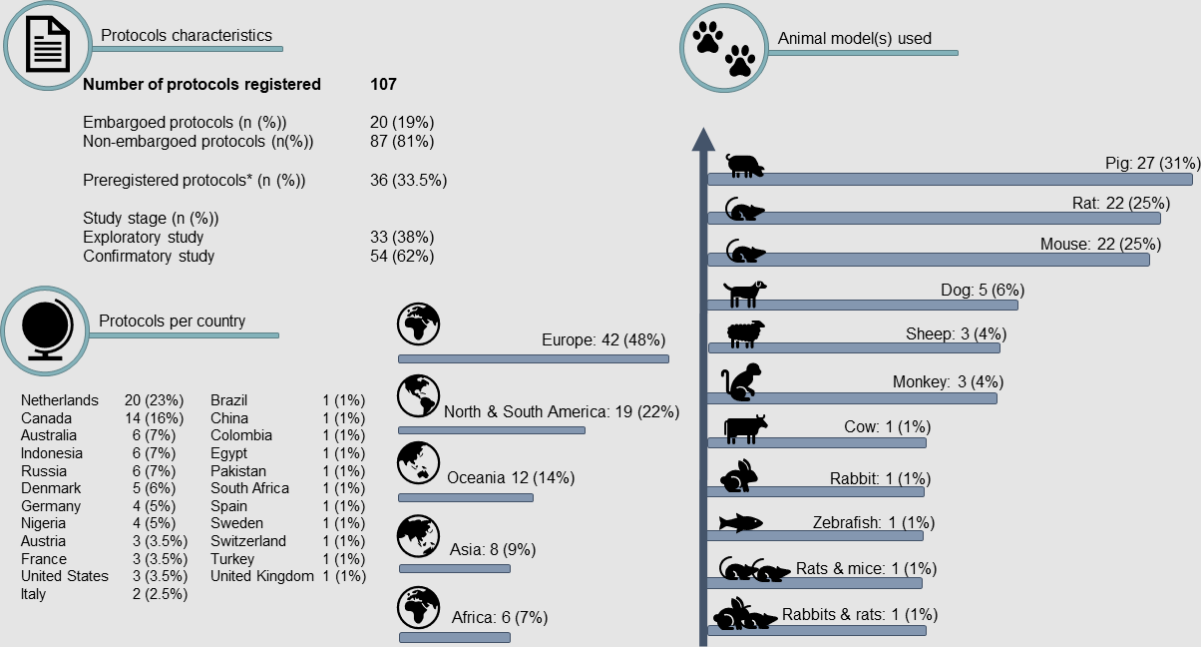
Protocols published on preclinicaltrials.eu on 20 January 2022. Note that only details of non-embargoed protocols are shown. *Preregistration is based on the reported study status at the first version of the submitted.

Three years after the official launch of PCT, there are over 1563 active accounts. Users originate from institutions in industry and academia in 30+ countries all over the world. Despite international recognition and encouraging engagement of stakeholders in, for example, the Netherlands, the number of registered protocols is still low.^32^ As of 20 January 2022, 107 protocols have been submitted, all of which have eventually been approved. The 87 non-embargoed protocols originate from 23 countries. They consist of both small animal (n=48, 55%) and large animal (n=39, 45%) studies and 54 studies (62%) are confirmatory studies (figure 2). Only a limited number of the overall protocols were registered before the start of the study (n=36, 33.5%). Of note, in January 2019, the German Centre for the Protection of Laboratory Animals (Bf3R) launched a comparable platform for registration of animal studies (www.animalstudyregistry.org).^33^ After 3 years, 102 studies from 14 different countries have been registered on this platform. Similarly, a low percentage of these studies was preregistered (n=21, 20.5%). Most studies are under embargo (n=81, 79.5%). Of the available non-embargoed protocols, 4 studies (19%) have a confirmatory character and 3 (14%) involve large animal models. Other platforms for preregistration exist, but they are not free of charge or do not focus primarily on animal research. In total, only 209 protocols have been registered on the dedicated animal study platforms over the last 3 years. Taking into account that over 58 million animals are used for scientific purposes globally, the amount of registered studies is still extremely low.^34^

## Preregistration of clinical trials

In comparison to preclinical registration, clinical trials registration is widely accepted and embraced by journals.^35^ The first clinical trial registries were established in the 1980s, mostly in the field of HIV–AIDS research.^36^ In 1989, the US government required the dissemination of information on HIV research, treatment and prevention, leading to the development of the AIDS Clinical Trials Information Service in 1989.^37^ In 1997, the US government required the National Institute of Health to provide a database of information on clinical trials for drugs for serious or life-threatening diseases and conditions, which resulted in the launch of clinicaltrials.gov in 2000.^38 39^ In 2005, the International Committee of Medical Journal Editors required all clinical trials to be registered in a public trial registry as a requirement for publication,^35^ resulting in an increasing number of trial registration.^40^ Over time, more than 15 clinical trial registries have arisen, which prompted the WHO to establish the International Clinical Trials Registry Platform (WHO ICTRP), a meta-search engine that allows searching through individual clinical trials registries. Over the years, clinical trial registries have provided us with opportunities for meta-research, for example, by providing insight into the frequency of reporting bias.^20 41^ These initiatives have been instrumental to improve the quality of biomedical research. Also, clinical trial registries are regularly searched for systematic reviews and provide additional data for meta-analysis.^42^

Following the example set by clinical trials registries, we may speculate that preclinical registration would need incentives from journal editors or governmental agencies to encourage researchers to preregister their studies.

## The future of preclinical preregistration

Preregistration might not be the only approach for improving translational research, but it is generally an easily implemented solution that will contribute to addressing various problems that currently reduce the impact of translational research. Ideally, preregistration would not be limited at all (eg, by an embargo), but the provided solutions act to lower the threshold for stakeholders to embrace preregistration and are therefore necessary at this phase of preregistration. We have learnt from our experiences so far and are continuously working on improving the platform. At this point, two free and public databases dedicated to preclinical registration exist, but this could increase, as has been the case for clinical trial registration platforms. A meta-search engine, like the WHO ICTRP, could be an added value for researchers. To carry our ambitions and increase the number of registered protocols, we designed a strategy for focusing on three main action points: promote, facilitate and understand. To promote preregistration among researchers, we will provide webinars and aim to develop e-learning tools. We also create promotional material and publish relevant information online, in collaborators’ newsletters or via short communications.^1^ Institutions, animal ethics committees and animal welfare bodies will be approached to aid in promoting preregistration and reward good behaviour. They can educate and encourage researchers to preregister their protocols. For this purpose, we recently developed a short video explaining preregistration (PCT).^43^

Moreover, an international ambassador network was started to further promote preregistration worldwide. Ambassadors commit to showing the example by preregistering themselves, promoting preregistration in their teams and institute and helping us reach out to important stakeholders in their countries.

To facilitate and ease preregistration, we are currently focusing on minimising the administrative burden for researchers.^44 45^ The obvious step to link data from locally required protocols to PCT should be further developed. Moreover, we will provide personal guidance with protocol registration when requested. To better understand stakeholders, we aim to gain knowledge about current practice and evaluate experiences with PCT. In collaboration with the University of Sydney, we are currently working on a survey among researchers on the believed benefits and concerns of preregistration. The results will provide us with additional information on how to improve the motivation of researchers to preregister. We will continue to discuss issues on preregistration and PCT with relevant stakeholders and evaluate the platform if necessary. In addition to this bottom-up approach, several stakeholders play a pivotal role in a top-down approach for the implementation of preregistration. Funders can guard quality in research by making preregistration mandatory for provided funding and journals can stimulate preregistration by setting it as a requirement for publication, just like they did for clinical preregistration.^35^ Committees and institutions involved in animal research can require accountability of previously provided animals as part of a new application. Journals can reward researchers who preregister, for example, with preregistration badges that are currently implemented by *BMJ Open Science* and the *Journal of Neuroscience Research* among others.^46 47^ In addition, journals play an important role in monitoring compliance. Institutes and funders can stimulate preregistration by incorporating preregistration in their reward system and monitor compliance by reviewing preregistration in applications.

## Concluding remarks

Preregistration increases transparency and contributes to more effective preclinical research. Multiple platforms to facilitate preregistration have been developed, but the number of registered protocols is still low. We show in this paper the development of and considerations behind PCT and highlight the growing interest for preregistration of animal studies and the role of multiple stakeholders in this endeavour. Several Dutch stakeholders have taken the lead in implementing preregistration. We are encouraging other stakeholders to follow these examples and thereby increase the number of registered protocols. At the same time, we keep putting preregistration on the agenda in all our discussions with relevant stakeholders. We believe it is time for the scientific community to take responsibility and move towards more effective animal research.

## Data Availability

All data relevant to the study are included in the article or uploaded as supplementary information. All data about the number of (pre)registered protocols and preregistration platforms are available freely at www.preclinicaltrials.eu (protocols data in full when creating an account) and at www.animalstudyregistry.org.

## References

[1] van der Naald M, Chamuleau SAJ, Menon JML, et al. A 3-year evaluation of preclinicaltrials.eu reveals room for improvement in preregistration of animal studies. PLoS Biol 2021;19:e3001397. 10.1371/journal.pbio.3001397 34499640 PMC8454931

[2] Steedman M, Taylor K, Stockbridge M. Unlocking productivity R&D productivity: Measuring the return of from pharmaceutical innovation, 2018.

[3] Kola I, Landis J. Can the pharmaceutical industry reduce attrition rates? Nat Rev Drug Discov 2004;3:711–6. 10.1038/nrd1470 15286737

[4] Freedman LP, Cockburn IM, Simcoe TS. The economics of reproducibility in preclinical research. PLoS Biol 2015;13:e1002165. 10.1371/journal.pbio.1002165 26057340 PMC4461318

[5] Leenaars C, Stafleu F, de Jong D, et al. A systematic review comparing experimental design of animal and human methotrexate efficacy studies for rheumatoid arthritis: lessons for the translational value of animal studies. Animals 2020;10. 10.3390/ani10061047. [Epub ahead of print: 17 06 2020]. PMC7341304 32560528

[6] Hooijmans CR, Leenaars M, Ritskes-Hoitinga M. A gold standard publication checklist to improve the quality of animal studies, to fully integrate the three RS, and to make systematic reviews more feasible. Altern Lab Anim 2010;38:167–82. 10.1177/026119291003800208 20507187

[7] Percie du Sert N, Hurst V, Ahluwalia A, et al. The ARRIVE guidelines 2.0: updated guidelines for reporting animal research. BMJ Open Sci 2020;4:e100115. 10.1136/bmjos-2020-100115 PMC7610906 34095516

[8] Ramirez FD, Motazedian P, Jung RG, et al. Methodological rigor in preclinical cardiovascular studies: targets to enhance reproducibility and promote research translation. Circ Res 2017;120:1916–26. 10.1161/CIRCRESAHA.117.310628 28373349 PMC5466021

[9] Wever KE, Hooijmans CR, Riksen NP, et al. Determinants of the efficacy of cardiac ischemic preconditioning: a systematic review and meta-analysis of animal studies. PLoS One 2015;10:e0142021. 10.1371/journal.pone.0142021 26580958 PMC4651366

[10] Jonker SJ, Menting TP, Warlé MC, et al. Preclinical evidence for the efficacy of ischemic postconditioning against renal ischemia-reperfusion injury, a systematic review and meta-analysis. PLoS One 2016;11:e0150863. 10.1371/journal.pone.0150863 26963819 PMC4786316

[11] Hesen NA, Riksen NP, Aalders B, et al. A systematic review and meta-analysis of the protective effects of metformin in experimental myocardial infarction. PLoS One 2017;12:e0183664. 10.1371/journal.pone.0183664 28832637 PMC5568412

[12] Crossley NA, Sena E, Goehler J, et al. Empirical evidence of bias in the design of experimental stroke studies: a metaepidemiologic approach. Stroke 2008;39:929–34. 10.1161/STROKEAHA.107.498725 18239164

[13] Hirst TC, Vesterinen HM, Conlin S, et al. A systematic review and meta-analysis of gene therapy in animal models of cerebral glioma: why did promise not translate to human therapy? Evid Based Preclin Med 2014;1:e00006:21–33. 10.1002/ebm2.6 27668084 PMC5020579

[14] van der Naald M, Wenker S, Doevendans PA, et al. Publication rate in preclinical research: a plea for preregistration. BMJ Open Sci 2020;4:e100051. 10.1136/bmjos-2019-100051 PMC8647586 35047690

[15] Wieschowski S, Biernot S, Deutsch S, et al. Publication rates in animal research. extent and characteristics of published and non-published animal studies followed up at two German University medical centres. PLoS One 2019;14:e0223758. 10.1371/journal.pone.0223758 31770377 PMC6879110

[16] ter Riet G, Korevaar DA, Leenaars M, et al. Publication bias in laboratory animal research: a survey on magnitude, drivers, consequences and potential solutions. PLoS One 2012;7:e43404. 10.1371/journal.pone.0043404 22957028 PMC3434185

[17] Macleod MR, O'Collins T, Howells DW, et al. Pooling of animal experimental data reveals influence of study design and publication bias. Stroke 2004;35:1203–8. 10.1161/01.STR.0000125719.25853.20 15060322

[18] Sena ES, van der Worp HB, Bath PMW, et al. Publication bias in reports of animal stroke studies leads to major overstatement of efficacy. PLoS Biol 2010;8:e1000344. 10.1371/journal.pbio.1000344 20361022 PMC2846857

[19] Perrin S. Preclinical research: make mouse studies work. Nature 2014;507:423–5. 10.1038/507423a 24678540

[20] Goldacre B, Drysdale H, Dale A, et al. Compare: a prospective cohort study correcting and monitoring 58 misreported trials in real time. Trials 2019;20:118. 10.1186/s13063-019-3173-2 30760329 PMC6375128

[21] Chamuleau SAJ, van der Naald M, Climent AM, et al. Translational research in cardiovascular repair: a call for a paradigm shift. Circ Res 2018;122:310–8. 10.1161/CIRCRESAHA.117.311565 29348252

[22] Kimmelman J, Anderson JA. Should preclinical studies be registered? Nat Biotechnol 2012;30:488–9. 10.1038/nbt.2261 22678379 PMC4516408

[23] Smith AJ. Guidelines for planning and conducting high-quality research and testing on animals. Lab Anim Res 2020;36:21. 10.1186/s42826-020-00054-0 32665911 PMC7348107

[24] Jansen of Lorkeers SJ, Doevendans PA, Chamuleau SAJ. All preclinical trials should be registered in advance in an online registry. Eur J Clin Invest 2014;44:891–2. 10.1111/eci.12299 25041644

[25] Netherlands Heart Institute Homepage [Internet], 2020. Available: www.heart-institute.nl/index.php?pagina=News%25id=45

[26] Royal Netherlands Academy of Arts and Sciences. Replication studies - Improving reproducibility in the emperical sciences, 2018.

[27] Groot D, Dik-Faber VM. Over Het registreren van alle individuele dierproeven: Motie van Het lid de Groot c.s, 2018: ISSN 0921-7371.

[28] Schouten C. Brief van de Minister van landbouw, natuur en voedselkwaliteit, 2019: ISSN 0921-7371.

[29] Engelshoven van. Pilot stimulering transparant proefdieronderzoek en voortgang van transitie naar minder proeven Met apen en meer proefdiervrije innovaties in Het BRPC, 2020.

[30] Kimmelman J, Mogil JS, Dirnagl U. Distinguishing between exploratory and confirmatory preclinical research will improve translation. PLoS Biol 2014;12:e1001863. 10.1371/journal.pbio.1001863 24844265 PMC4028181

[31] ZonMw. Transparency on research projects, findings and data [Internet]. Available: https://www.zonmw.nl/en/research-and-results/fair-data-and-data-management/2-transparency-on-research-projects-findings-and-data/

[32] Baker M. Animal registries aim to reduce bias. Nature 2019;573:297–8. 10.1038/d41586-019-02676-4 31501583

[33] Bert B, Heinl C, Chmielewska J, et al. Refining animal research: the animal study registry. PLoS Biol 2019;17:e3000463. 10.1371/journal.pbio.3000463 31613875 PMC6793840

[34] Taylor K, Gordon N, Langley G, et al. Estimates for worldwide laboratory animal use in 2005. Altern Lab Anim 2008;36:327–42. 10.1177/026119290803600310 18662096

[35] De Angelis C, Drazen JM, Frizelle FA, et al. Clinical trial registration: a statement from the International Committee of medical Journal editors. N Engl J Med 2004;351:1250–1. 10.1056/NEJMe048225 15356289

[36] Easterbrook PJ. Directory of registries of clinical trials. Stat Med 1992;11:345–423. 10.1002/sim.4780110307 1609175

[37] Katz DG, Dutcher GA, Toigo TA, et al. The AIDS clinical trials information service (ACTIS): a decade of providing clinical trials information. Public Health Rep 2002;117:123–30. 10.1016/S0033-3549(04)50118-8 12356996 PMC1497425

[38] US Government. Food and drug administration modernization act (FDAMA) of 1997, 1997.

[39] clinicaltrials.gov homepage [Internet], 2021. Available: https://www.clinicaltrials.gov/

[40] Zarin DA, Tse T, Ide NC. Trial registration at ClinicalTrials.gov between may and October 2005. N Engl J Med 2005;353:2779–87. 10.1056/NEJMsa053234 16382064 PMC1568386

[41] DeVito NJ, Bacon S, Goldacre B. Compliance with legal requirement to report clinical trial results on ClinicalTrials.gov: a cohort study. Lancet 2020;395:361–9. 10.1016/S0140-6736(19)33220-9 31958402

[42] Baudard M, Yavchitz A, Ravaud P, et al. Impact of searching clinical trial registries in systematic reviews of pharmaceutical treatments: methodological systematic review and reanalysis of meta-analyses. BMJ 2017;356:j448. 10.1136/bmj.j448 28213479 PMC5421496

[43] preclinicatrials.eu [Internet], 2021. Available: https://preclinicaltrials.eu/

[44] Wieschowski S, Silva DS, Strech D. Animal study registries: results from a Stakeholder analysis on potential strengths, weaknesses, facilitators, and barriers. PLoS Biol 2016;14:e2000391. 10.1371/journal.pbio.2000391 27832101 PMC5104355

[45] Wieschowski S, Laser H, Sena ES, et al. Attitudes towards animal study registries and their characteristics: an online survey of three cohorts of animal researchers. PLoS One 2020;15:e0226443. 10.1371/journal.pone.0226443 31905203 PMC6944338

[46] Journal of Neuroscience Research. Preregistered badge. Available: https://onlinelibrary.wiley.com/page/journal/10974547/homepage/open_research_badges.html

[47] BMJ Open Science. Preregistered badge BMJ Open Science [Internet]. Available: https://openscience.bmj.com/pages/policies/#Ethics

